# Supplementary material for: Uric acid to HDL cholesterol ratio as a novel predictor of carotid intima-media thickness: a cross-sectional study in rural China
Source: PeerJ. 2025 Sep 19;13:e20053. doi: 10.7717/peerj.20053 (PMC12452943; doi:10.7717/peerj.20053)
Supplement: Supplemental Information 1 [file peerj-13-20053-s001.docx]

Supplementary Table 1. Univariate general linear model analysis of factors affecting IMT

| Item | β (95% CI) | Robust SE | P |
| --- | --- | --- | --- |
| UHR | 0.09 (0.05, 0.14) | 0.024 | ＜0.001 |
| Gender | -41.53 (-51.31, -31.76) | 4.985 | ＜0.001 |
| Age | 6.15 (5.59, 6.71) | 0.286 | ＜0.001 |
| Hypertension | 44.49 (33.54 55.44) | 5.584 | ＜0.001 |
| Diabetes | 24.17 (11.71, 36.62) | 6.352 | ＜0.001 |
| Smoking | 40.31 (30.36, 50.26) | 5.075 | ＜0.001 |
| Alcohol consumption | 27.55 (17.24, 37.86) | 5.259 | ＜0.001 |
| BMI | -0.83 (-2.13, 0.48) | 0.667 | 0.216 |
| Systolic blood pressure | 1.45 (1.20, 1.70) | 0.126 | ＜0.001 |
| Diastolic blood pressure | -0.01 (-0.44, 0.43) | 0.223 | 0.982 |
| Differential pulse pressure | 2.24 (1.92, 2.55) | 0.162 | ＜0.001 |
| GLU | 6.69 (3.85, 9.53) | 1.448 | ＜0.001 |
| TC | 6.27 (1.01, 11.52) | 2.680 | 0.019 |
| TG | -2.09 (-5.68, 1.50) | 1.830 | 0.252 |
| HDL-C | -11.06 (-24.57, 2.44) | 6.888 | 0.108 |
| LDL-C | 7.61 (1.66, 13.55) | 3.033 | 0.012 |
| SUA | 0.12 (0.06, 0.18) | 0.030 | ＜0.001 |

Table Note: GLU, TC, TG, LDL-C, HDL-C are in mmol/L, and uric acid is in μmol/L. "Robust SE" refers to robust standard errors.
