# Supplementary material for: Uric acid to HDL cholesterol ratio as a novel predictor of carotid intima-media thickness: a cross-sectional study in rural China
Source: PeerJ. 2025 Sep 19;13:e20053. doi: 10.7717/peerj.20053 (PMC12452943; doi:10.7717/peerj.20053)
Supplement: Supplemental Information 2 [file peerj-13-20053-s002.docx]

Supplementary Table 2. Univariate analysis of factors affecting carotid intima-media thickening

| Item | OR (95% CI) | P |
| --- | --- | --- |
| UHR | 1.002 (1.001, 1.004) | ＜0.001 |
| Gender | 0.32 (0.22, 0.48) | ＜0.001 |
| Age | 1.09 (1.07, 1.12) | ＜0.001 |
| Age groups |  | ＜0.001 |
| ＜60 | reference | reference |
| 60-70 | 4.43 (2.01, 9.76) | ＜0.001 |
| ≥70 | 10.70 (4.87, 23.53) | ＜0.001 |
| Smoking | 2.48 (1.71, 3.60) | ＜0.001 |
| Alcohol consumption | 2.13 (1.49, 3.06) | ＜0.001 |
| Hypertension | 2.59 (1.45, 4.63) | 0.001 |
| Diabetes | 1.57 (1.05, 2.35) | 0.029 |
| BMI | 1.02 (0.97, 1.07) | 0.404 |
| Systolic blood pressure | 1.03 (1.02, 1.04) | ＜0.001 |
| Diastolic blood pressure | 1.01 (0.99, 1.03) | 0.202 |
| Differential pulse pressure | 1.04 (1.03, 1,05) | ＜0.001 |
| GLU | 1.10 (1.02, 1.20) | 0.021 |
| TC | 1.17 (0.98, 1.40) | 0.088 |
| TG | 0.97 (0.84, 1.13) | 0.705 |
| HDL-C | 0.75 (0.45, 1.27) | 0.285 |
| LDL-C | 1.15 (1.00, 1.33) | 0.054 |
| SUA | 1.004 (1.002, 1.005) | ＜0.001 |

Table Note: GLU, TC, TG, LDL-C, HDL-C are in mmol/L, and uric acid is in μmol/L.
