# Supplementary material for: Uric acid to HDL cholesterol ratio as a novel predictor of carotid intima-media thickness: a cross-sectional study in rural China
Source: PeerJ. 2025 Sep 19;13:e20053. doi: 10.7717/peerj.20053 (PMC12452943; doi:10.7717/peerj.20053)
Supplement: Supplemental Information 3 [file peerj-13-20053-s003.docx]

Supplementary Table 3. Univariate analysis of UHR and carotid intima-media thickening in elderly men

| Item | OR (95% CI) | P |
| --- | --- | --- |
| UHR | 1.003 (1.000, 1.004) | 0.004 |
| Smoking | 1.15 (0.61, 2.17) | 0.658 |
| Alcohol consumption | 0.93 (0.56, 1.55) | 0.782 |
| Hypertension | 1.60 (0.83, 3.06) | 0.161 |
| Diabetes | 2.06 (1.22, 3.49) | 0.007 |
| BMI | 1.12 (1.05, 1.19) | ＜0.001 |
| Systolic blood pressure | 1.02 (1.01, 1.03) | 0.003 |
| Diastolic blood pressure | 1.00 (0.98, 1.02) | 0.719 |
| Differential pulse pressure | 1.03 (1.01, 1.04) | <0.001 |
| GLU | 1.11 (1.00, 1.23) | 0.055 |
| TC | 1.37 (1.01, 1.73) | 0.008 |
| TG | 1.06 (0.95, 1.18) | 0.311 |
| HDL-C | 0.52 (0.28, 1.00) | 0.049 |
| LDL-C | 1.50 (1.13, 1.97) | 0.004 |
| SUA | 1.003 (1.000, 1.005) | 0.032 |

Table Note: GLU, TC, TG, LDL-C, HDL-C are in mmol/L, and uric acid is in μmol/L.
