# Supplementary material for: Uric acid to HDL cholesterol ratio as a novel predictor of carotid intima-media thickness: a cross-sectional study in rural China
Source: PeerJ. 2025 Sep 19;13:e20053. doi: 10.7717/peerj.20053 (PMC12452943; doi:10.7717/peerj.20053)
Supplement: Supplemental Information 6 [file peerj-13-20053-s006.doc]

STROBE Statement—Checklist of items that should be included in reports of ***cross-sectional studies***

|  | Item No | Recommendation |
| --- | --- | --- |
| **Title and abstract** | 1 | (*a*) 42-45 |
|  |  | (*b*) 65-68 |
| Introduction | | |
| Background/rationale | 2 | 72-101 |
| Objectives | 3 | 102-106 |
| Methods | | |
| Study design | 4 | 109-117 |
| Setting | 5 | 109-110 |
| Participants | 6 | (*a*)110-114 |
| Variables | 7 | 120-153 |
| Data sources/ measurement | 8* | 120-153 |
| Bias | 9 | Describe any efforts to address potential sources of bias |
| Study size | 10 | Explain how the study size was arrived at |
| Quantitative variables | 11 | 120-153 |
| Statistical methods | 12 | (*a*) 155-165 |
|  |  | (*b*) 158-163 |
|  |  | (*c*) Explain how missing data were addressed |
|  |  | (*d*) If applicable, describe analytical methods taking account of sampling strategy |
|  |  | (*e*) Describe any sensitivity analyses |
| Results | | |
| Participants | 13* | (a) 167-170 |
|  |  | (b) 167-170 |
|  |  | (c) 167-170 |
| Descriptive data | 14* | (a) 172-176 |
|  |  | (b) Table 1 |
| Outcome data | 15* | 172-176 |
| Main results | 16 | (*a*) 178-188 |
|  |  | (*b*)139-141 |
|  |  | (*c*) If relevant, consider translating estimates of relative risk into absolute risk for a meaningful time period |
| Other analyses | 17 | 201-207 |
| Discussion | | |
| Key results | 18 | 213-226 |
| Limitations | 19 | 253-271 |
| Interpretation | 20 | 227-252 |
| Generalisability | 21 | Discuss the generalisability (external validity) of the study results |
| Other information | | |
| Funding | 22 | 288-298 |

*Give information separately for exposed and unexposed groups.

**Note:** An Explanation and Elaboration article discusses each checklist item and gives methodological background and published examples of transparent reporting. The STROBE checklist is best used in conjunction with this article (freely available on the Web sites of PLoS Medicine at http://www.plosmedicine.org/, Annals of Internal Medicine at http://www.annals.org/, and Epidemiology at http://www.epidem.com/). Information on the STROBE Initiative is available at www.strobe-statement.org.
